# What is in a food store name? Leveraging large language models to enhance food environment data

**DOI:** 10.3389/frai.2024.1476950

**Published:** 2024-12-06

**Authors:** Analee J. Etheredge, Samuel Hosmer, Aldo Crossa, Rachel Suss, Mark Torrey

**Affiliations:** Center for Population Health Data Science, NYC Department of Health and Mental Hygiene, New York City, NY, United States

**Keywords:** natural language processing, deep learning, machine learning, food environment classification, administrative food data, health department, food store name, large language models

## Abstract

**Introduction:**

It is not uncommon to repurpose administrative food data to create food environment datasets in the health department and research settings; however, the available administrative data are rarely categorized in a way that supports meaningful insight or action, and ground-truthing or manually reviewing an entire city or neighborhood is rate-limiting to essential operations and analysis. We show that such categorizations should be viewed as a classification problem well addressed by recent advances in natural language processing and deep learning—with the advent of large language models (LLMs).

**Methods:**

To demonstrate how to automate the process of categorizing food stores, we use the foundation model BERT to give a first approximation to such categorizations: a best guess by store name. First, 10 food retail classes were developed to comprehensively categorize food store types from a public health perspective.

**Results:**

Based on this rubric, the model was tuned and evaluated (F1_micro_ = 0.710, F1_macro_ = 0.709) on an extensive storefront directory of New York City. Second, the model was applied to infer insights from a large, unlabeled dataset using store names alone, aiming to replicate known temporospatial patterns. Finally, a complimentary application of the model as a data quality enhancement tool was demonstrated on a secondary, pre-labeled restaurant dataset.

**Discussion:**

This novel application of an LLM to the enumeration of the food environment allowed for marked gains in efficiency compared to manual, in-person methods, addressing a known challenge to research and operations in a local health department.

## 1 Introduction

The food environment, a term that broadly captures the “physical, economic, political, and socio-cultural context in which consumers engage with the food system to make their decisions about acquiring, preparing and consuming food” (HLPE, 2017), is upstream of many inequitable health outcomes and is challenging to study in its complex and dynamic forms (Boise et al., 2023). Sustained disinvestment in public health and the siloing of skills have prevented advancements required for government stakeholders to adequately address the food environment’s impact on food equity and disparate health outcomes (Brown et al., 2019). Much of the food environment data that are readily available to municipal agencies are from open administrative data sources on food licensing or inspections that require manual cleaning, categorization, merging, and ground-truthing to create a repurposed snapshot of the built food environment (Burgoine, 2010).

Food environment categories are most broadly defined by where they exist (e.g., natural or built environments) and the food acquisition behavior they elicit (e.g., shopping or dining) (Downs et al., 2020). More specifically, innate categories emerge that bear resemblance based on attributes (i.e., a shop could be a *Grocery* store if it consistently has a full range of foodstuffs that are safe to eat, at an expected price point, if using the attributes of availability, price, safety, and convenience to assign membership). Importantly, these attributes also apply to categories outside of grocery stores and demonstrate the need to allow for overlapping categories to exhaustively classify the food environment (in New York City, a “bodega” almost always sells some *groceries*) (Hirsch et al., 2021). Here, we see that store categories are determined by like attributes that can be overlapping—by so-called *family resemblances* (Wittgenstein, 1953; Halevy et al., 2009). What then makes the categories meaningful in a public health context are their intended uses. In epidemiology parlance, “meaningful categories” represent legitimate targets for intervention, as the categories act as proxies for a theorized exposure of coordinated actors upon a defined health outcome.

Store names not only signal to shoppers the nature of the goods on offer but are a feature common to all public food environment datasets^1^, both nationally and in New York City. A model that could learn from the NYC food environment by yielding a credible best guess as to which meaningful categories a store belongs, based on store name alone, would enhance these datasets with actionable information suited for epidemiologic study (Hirsch et al., 2021).

Over the last several decades, the field of NLP has been revolutionized by fitting as-large-as-possible models to as-large-as-possible-corpuses. Colloquially, such models are referred to as large language models (LLMs). The advent of the transformer architecture (Vaswani et al., 2017) and its task-agnostic descendants spawned many state-of-the-art deep learning LLMs across a variety of tasks in NLP, with models ever-often scaling in the number of parameters and (pre-)training examples (Brown et al., 2020). For this task, we focus on (namely classification) the bidirectional encoder representations from transformers (BERT), which is among the first transformer models to achieve state-of-the-art benchmarks on NLP datasets (Devlin et al., 2018) (see Section 2.4).

The success of transformers stems from two key properties of their constitution. The first is the attention mechanism, allowing for appropriate positioning of words in embedding space based on the words around them (Vaswani et al., 2017). The second is that deep learning models perform better (i.e., have lower generalization error) as the number of parameters grows in relation to the number of training examples (Nakkiran et al., 2021). Relatedly, sufficiently deep neural networks are more likely to find low-complexity targets (Mingard et al., 2021; Goldblum et al., 2023). In this way, large neural networks—and in particular transformers—are, contrary to popular belief, more prone to produce simple^2^ solutions to noisy problems (e.g., Goldblum et al. demonstrate this simplicity bias of GPT3 in an idealized setting). We view this tendency to be crucial, as our model’s best guess of what a food store is—by name alone—should be analogous to the heuristic intuition (Gigerenzer and Gaissmaier, 2011) of a native of the food environment in NYC, which might not differ all-that-much from the intuition of a food environment native in larger swaths of the United States.

LLMs address the costs and challenges of by-hand food environment classification through automation. To automate the arduous process of manually reviewing store records and meaningfully categorizing food stores, we used machine learning to give a first approximation to such categorizations: the best guess by store name. Building on the earlier use of LLMs to predict cuisine type based on retailer names in the United Kingdom (Bishop et al., 2021), a classifier was developed by fine-tuning a transformer on a snapshot of the NYC food environment.

The aims of this study were to (1) train and evaluate the performance of an LLM classifier on labeled food retail locations in NYC, (2) perform inference on a large unlabeled dataset using store name and replicate known temporospatial patterns, and (3) determine potential data quality enhancements for a secondary, pre-labeled restaurant dataset. Achieving or exceeding “good” performance based on the published threshold would indicate the viability of these methods for adoption in the longitudinal analysis of the food environment and as data quality enhancement (DQE) techniques for operational and research pipelines.

## 2 Materials and methods

A transformer LLM was trained and tested on labeled NYC storefront data based on the 10-item NYC food environment categories. These major food retail classes were developed from a public health perspective, drawing on previous studies (Cohen et al., 2020; Hirsch et al., 2021), and informed by the authors’ working contextual knowledge as New Yorkers (Table 1).

**Table 1:** Non-exclusive categories for the classification of the NYC food environment.

Food retail classes	Description of food store families
*Specialty Foods*	Goods stores that predominantly sell only a specific type of foods such as cheese, meat, fish, or fruits, health food (e.g., supplements), and international markets
*Convenience*	Smaller goods stores that include beverage coolers, lotto/candy, and common pantry items
*Fast Food*	Includes places providing the service of preparing standardized food items served or consumed quickly (e.g., chain fast food and deli counters)
*Discount*	Stores that sell items at a discount (e.g., 99 cents) and generally do not sell fresh food
*Grocery*	Goods stores offering a full range of groceries, including smaller corner groceries and supermarkets
*Restaurant*	Includes places providing the service of preparing food items consumed on-premises usually with table service
*Alcohol Store*	Goods stores where alcohol is the primary offering
*Alcohol Bar*	Includes locations with bars dedicated to the serving of alcohol on premises
*Juice/Coffee Shops*	Stores serving coffee, tea, smoothies, or juice
*Sweets and Desserts*	Includes sweets and dessert stores, and bakeries that also sell desserts

### 2.1 Data acquisition

#### 2.1.1 Labeled data

Training and validation datasets were assembled from a private retail directory created and maintained by Live XYZ and licensed through the City of New York. The directory snapshot, obtained in September 2023, consisted of all currently operating retail store locations in NYC at the time of the pull. This directory is regularly ground-truthed by Live XYZ surveyors according to a retail store taxonomy developed by the company. The conversion from their store taxonomy to our 10-food environment categories is outlined in Section 2.2.1 and 2.3.

#### 2.1.2 Inference data

The proposed inference dataset was acquired as public use data from the New York State Agriculture and Markets (NYSAM) Office. Longitudinal data were obtained through a Freedom of Information Act request for data covering the period of January 2012 through December 2023. The NYSAM office maintains a database of food retail locations that are licensed and inspected by New York State, including only food retail locations where <50% of their sales revenue is from prepared food. Note that retail food locations within NYC where >50% of their sales are from prepared food (e.g., restaurants) are inspected by the NYC Department of Health and Mental Hygiene and are excluded from the NYSAM database. Key columns in this dataset are store name, location, and date of inspection.

The NYC Health Department is responsible for inspecting prepared food service establishments and maintains an extensive longitudinal dataset of restaurants from inspection records. Food retailers with >50% of their sales from prepared food are instead inspected by the NYC Health Department and are therefore represented in the Restaurants data. As part of the permitting process, service description tags are retained in the data and can roughly be apportioned into the classifier’s categories of *Sit-down Restaurant* and *Fast Food*. These data were selected as another inference data source to evaluate the DQE capabilities of the classifier given the presence of the common feature (store name) and an extant (though independent) tag set. Key columns in this dataset are store name, location, service description, venue, and date of inspection.

Additionally, Live XYZ shared out-of-state snapshots, which were used as a loose estimate of model error through time due to drift. In summary, Live XYZ data were used to test and train the model, while the NYSAM and NYC Restaurants administrative food store data were selected as the inference datasets.

### 2.2 Data cleaning and preprocessing

#### 2.2.1 Labeled data

A dataset of food retail store names and labels was processed from the Live XYZ directory by first restricting this snapshot of retail stores to the meta store categories ‘Food’, ‘Essentials’, or ‘Drink’. Subcategories such as ‘Restaurant’ and ‘Grocery & Convenience’ were refined by more granular tags present in the data. For example, an item was given the final label *grocery* if it had the tag ‘supermarket’ or ‘ *Grocery* store’ but not if it only had the tag ‘convenience store.’ The ‘convenience store’ tag was present in pharmacies and gas stations where such capacity exists (e.g., Duane Reade’s and Shell Food Marts). For the subcategory ‘restaurant’ several tags were grouped and included in the *Sit-down Restaurant* (henceforth abbreviated to *Restaurant*) or *Fast Food* categories depending on whether the Live XYZ surveyors had flagged the establishment as being ‘dine-in’ and/or ‘quick-bites’. For example, stores with the tags ‘kosher delicatessen’, ‘bagel shop’, ‘pizzeria’, ‘taqueria’, ‘French cafe’, ‘diner’, ‘deli’, ‘sandwich shop’, ‘burger joint’, ‘gyro shop’, and ‘fried chicken restaurant,’ among others, were only categorized as *Fast Food* or *Restaurant* depending on the presence of the ‘quick bites’ or ‘dine-in’ flags, respectively. If both flags were present, the store would contain both labels *Fast Food* and *Restaurant.*

To avoid leakage, the data were deduplicated on the store name. Labels were selected based on higher-order modal venue categorizations (i.e., whatever categorizations a fixed food store had most frequently across all multi-labels), with tie-breaks going to the most recently surveyed establishments. For example, any store named exactly “John’s” would be given the multi-label *Alcohol Bar* and *Restaurant* if these were the most frequently occurring multi-labels paired with “John’s” in the dataset. If “John’s” were a multi-labeled-only *Restaurant,* as many times as it was a multi-labeled *Alcohol Bar*, *Restaurant* then the most recent surveyor labeling would break the tie. The final dataset of 24,901 food stores was then split into 50% training, 25% validation, and 25% test datasets. Exploiting the trade-off of low-generalization-error with memorization (Zhang et al., 2021) stores with more than 10 locations across the 5-boroughs were set aside from the train-test split and later added back into the training set so that they would be memorized. As none of the memorized stores showed up in the test, our performance scores reflect a lower bound on the true performance of the model at inference.

Finally, changes in the NYC food environment that might negatively impact model performance globally through time were evaluated by inferencing an alternate geography also using name alone. To facilitate this aim, Live XYZ provided pre-released non-NYC directory data. The San Francisco Bay area was selected for prominent similarities in the density of population, affordability of housing, and socioeconomic disparities, as well as salient regulatory discrepancies that would bias the classifier—chiefly in the overlap between alcohol stores and grocery stores. All of the above preprocessing steps were applied to create a new test set from the San Francisco Bay area geography. The final preprocessed dataset consisted of only 9,185 labeled food stores with name duplication included.

#### 2.2.2 Inference data

The NYSAM longitudinal data were geocoded using version 21B of the NYC Department of City Planning’s Geosupport geocoding software. During this process, addresses were cleaned and retail food locations outside of NYC were excluded. Additional efforts were made to correct addresses, as previously described by Greene et al. (2023). The final inference dataset had 33,964 unique point-located storefronts. Evident dates of operation were determined by inspection dates and the recorded out-of-business date.

The NYC Health Department Restaurants inspection dataset initially consisted of 68,135 unique storefronts; 2,329 were dropped due to mixed datatypes and null values, resulting in a final dataset of 65,806 unique storefronts. The key variables in the dataset are food store descriptors (including cuisine type, service description, and venue tags) and geographic information (point location and address).

### 2.3 Classification by food store name

The classifier was premised on 10 NYC food environment categories, which are roughly ontologically divided between goods and services and are, importantly, not mutually exclusive. For example, stores classified as both *Convenience* and *Fast Food* would be what NYC residents would describe as a bodega. To illustrate how the model would select classes by way of example, if the store ‘Oak and Steel’ is taken to be a food store, the most intuitively fitting labels applicable should be assigned independently to each (e.g., in this case perhaps just Alcohol Store, Alcohol Bar, or both). The food store ‘La Vina Deli Grocery’ is heuristically more likely to be a *Convenience* store with a deli counter (*Fast Food*) than it is to be a *Grocery*, and ‘Butcher Bar’ is heuristically more likely to be a *Restaurant* with an *Alcohol Bar* than it is to be a butcher shop.

The categories of stores were the following: *Specialty Foods*, *Convenience*, *Fast Food*, *Discount*, *Grocery*, *Restaurant*, *Alcohol Store*, *Alcohol Bar*, *Juice/Coffee*, and *Sweets and Desserts* (see Table 1). To illustrate the desired behavior of the classifier, the model would, for example, take as input the store names ‘La Vina Deli Grocery’ and ‘Butcher Bar’ and predict the best fitting multi-labels ‘*Convenience, Fast Food*’ and ‘*Alcohol Bar, Restaurant,*’ respectively.

### 2.4 Machine learning

The encoder model BERT was selected for the task of sentence classification. The original implementations of BERT varied by the number of repetitions of the encoder block in the model architecture (Devlin et al., 2018). The ‘Large’ version consists of 24 encoder blocks, resulting in approximately 350 million model parameters. BERT and subsequent variations were state-of-the-art on the Stanford Question Answering Dataset (SQUAD) and General Language Understanding and Evaluation (GLUE) datasets until 2022 (Rajpurkar et al., 2018; Wang et al., 2018).

The architecture and weights of the BERT-Large model were downloaded from the Hugging Face platform^3^ instead of acquiring the model via the Transformers API checkpoint to comply with the NYC Health Department infrastructure policy. The uncased model was chosen due to discrepancies in the capitalization of store names observed between our proposed inference datasets.

Python packages pandas, NumPy, and scikit-learn were used for data wrangling and preprocessing of both training data and inference. Hugging Face’s Transformers APIs in conjunction with deep-learning frameworks PyTorch and TensorFlow were used for tuning BERT-Large on the NYC Health Department’s Machine Learning Server (Nucleus) with NVIDIA A100 Tensor Core GPUs. The model was tuned using the Adam optimizer with a learning rate of $10^{-6}$ with $\beta_{1}=0.9$ and $\beta_{2}=0.999$ (Kingma and Ba, 2014). Finally, early stopping was used as a callback by monitoring macro-F1 (see 2.5.1) on the validation set^4^.

### 2.5 Performance metrics and geospatial methods

#### 2.5.1 Assessing classifier performance

The classifier’s performance was assessed by calculating the *precision* (also known as the positive predictive value) and the *recall* (also known as sensitivity) on the test set. The precision of a binary classifier is the ratio of true positives to predicted positives, or the true positive rate, whereas recall is the ratio of true positives to actual positives or the actual positive rate. Published sensitivity cutoffs for food environment classification were applied to our sensitivity analyses: < 20% very poor, 21–30% poor, 31–50% fair, 51–71% moderate, 71–90% good, and > 90% excellent (Paquet et al., 2008; Bishop et al., 2021). High precision with low recall would indicate that although actual positives are not well detected, one would still have confidence in the positives that are identified. Finally, the F1 score—the harmonic mean of precision and recall—was calculated per class.


$$F1=\frac{\mathrm{true}\;\mathrm{positives}\;}{\mathrm{true}\;\mathrm{positives}+½\left(\mathrm{false}\;\mathrm{positives}+\mathrm{false}\;\mathrm{negatives}\right)}$$

The two natural generalizations of the F1 score to the multi-class setting—micro and macro F1 scores—were also calculated as an assessment of overall model performance where, specifically, the macro F1 score is the arithmetic mean of the per-class F1 scores, and the micro F1 score is the above true positive ratio obtained by aggregating true and false positives as well as false negatives across all classes.

In summary, precision and recall are metrics that, respectively, provide a measure of the success of the classifier in correctly predicting and detecting true positives. The F1 score is a combination of these metrics that is a popular measure of classification performance per category and can be generalized to evaluate overall performance in multi-class classification settings.

#### 2.5.2 Replicating known temporospatial patterns

To visualize the inference data, static hexbin maps were generated for all five boroughs to demonstrate the longitudinal nature of the data by showing the average change in retail type distributions. As a coarse recapitulation of the changes in the NYC food environment during the COVID-era described by Yi et al. (2022), average per-hexbin changes from 2019 to 2021 were calculated as the mean annual changes in store operation and closure status. Neigborhood Tabulation Area (NTA) boundaries (NYC, 2020) were overlaid by the half-mile hexbins (i.e., a representation of a 10-min walk from centroid to centroid); this custom geography visualization was chosen as a visual indicator of the modifiable aerial unit problems (MAUPs) common in food environment analyses (i.e., food acquisition behaviors are not guided by administrative boundaries) (Boise et al., 2023).

## 3 Results

### 3.1 Performance on hold-out test set

**Figure 1:**
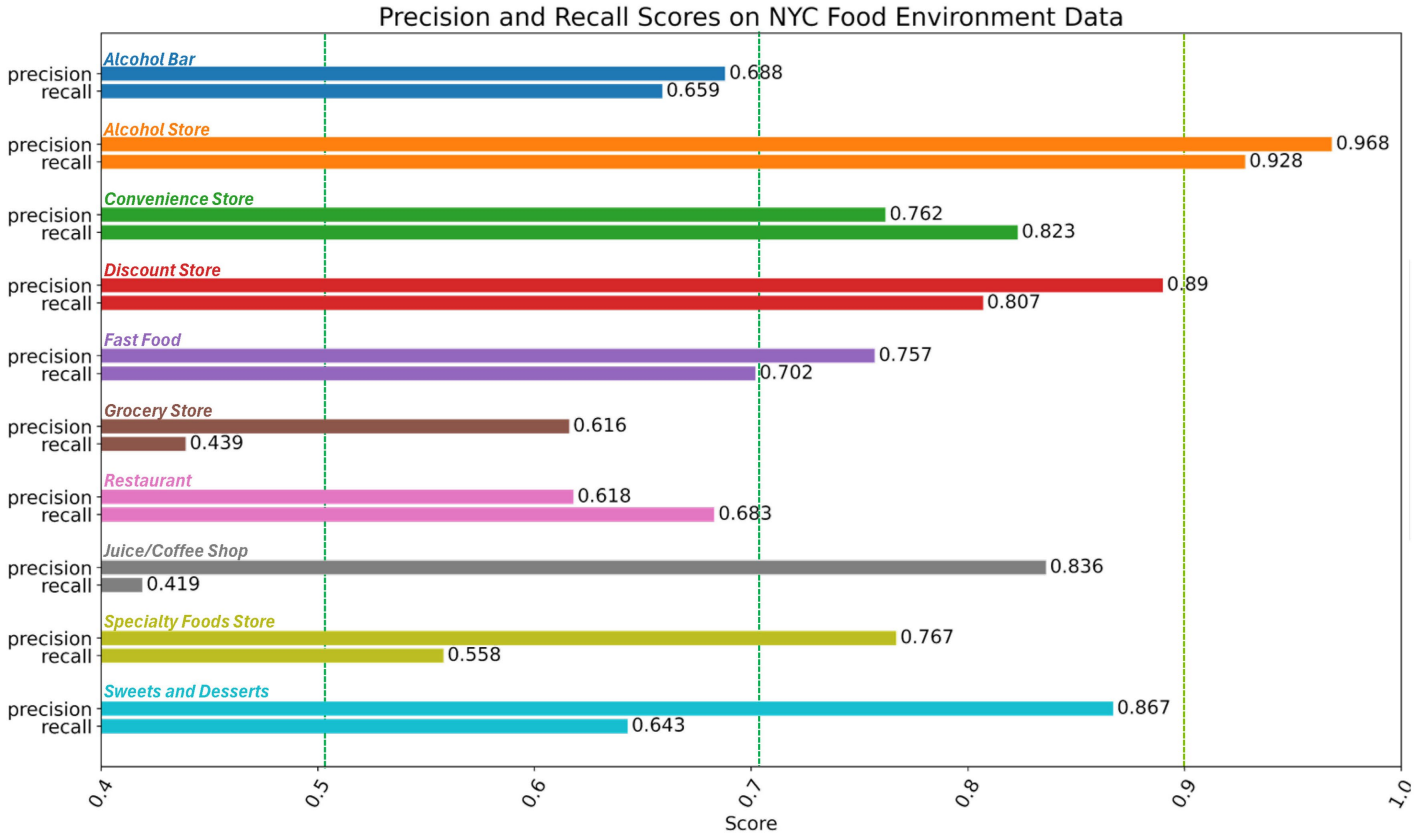
Sensitivity and recall for the hold-out test set. Dashed vertical lines show adopted sensitivity cutoffs for food environment classification were applied to our sensitivity analyses: <20% very poor, 21–30% poor, 31–50% fair, 51%–71 moderate, 71–90% good, and > 90% excellent (Paquet et al., 2008; Bishop et al., 2021).

The final version of the model had good performance according to the adopted food environment classifier performance cutoffs (F1_micro_ = 0.710, F1_macro_ = 0.709). The per-category performance metrics indicate that performance scores broadly represent the good and excellent performance ranges in both precision and recall (*Alcohol Store*, *Convenience Store*, and *Discount Store*) or good in precision alone (*Fast Food*, *Coffee/Juice Shop*, *Specialty Foods Store*, and *Sweets and Desserts*), with few scores falling within the moderate range in both precision and recall (*Alcohol Bar* and *Restaurant*). The *Grocery* category had the lowest F1 with only fair recall and moderate precision (Table 2; Figure 1). Table 3 demonstrates examples of model outputs on store name inputs mentioned in Section 2.3. The variable ‘input’ is assigned an example food store name and fed into the model. We present the Boolean and raw score predictions per class, where the Boolean value is determined by thresholding the raw score at 0.5.

**Table 2:** Heatmap of model performance metrics on the hold-out test set.

Store category	Precision	Recall	F1-Score
*Alcohol Bar*	0.688	0.659	0.673
*Alcohol Store*	0.968	0.928	0.947
*Convenience Store*	0.762	0.823	0.791
*Discount Store*	0.89	0.807	0.846
*Fast Food*	0.757	0.702	0.729
*Grocery Store*	0.616	0.439	0.513
*Restaurant*	0.618	0.683	0.649
*Coffee/Juice Shop*	0.836	0.419	0.559
*Specialty Foods Store*	0.767	0.558	0.646
*Sweets and Desserts*	0.867	0.643	0.738

Performance: 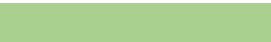 Excellent (>90%), 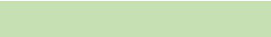 Good (71–90%), 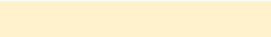 Moderate (51–71%), 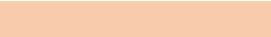 Fair (31–50%).

**Table 3:** Example outputs of the model on selected food retail stores.

	Input: “Oak and Steel”		Input: “La Vina Deli Grocery”		Input: “Butcher Bar”	
Food retail class	Boolean	Raw score	Boolean	Raw score	Boolean	Raw score
Alcohol bar	**True**	**0.688**	False	0.006	**True**	**0.797**
Alcohol store	False	0.406	False	0.003	False	0.010
Convenience Store	False	0.057	**True**	**0.885**	False	0.007
Discount Store	False	0.023	False	0.003	False	0.003
Fast Food	False	0.025	**True**	**0.942**	False	0.019
Grocery Store	False	0.018	False	0.069	False	0.011
Restaurant	False	0.411	False	0.009	**True**	**0.597**
Coffee/Juice Shop	False	0.075	False	0.008	False	0.018
Specialty Foods Store	False	0.031	False	0.038	False	0.041
Sweets and Desserts	False	0.009	False	0.008	False	0.011

Values exceeding the threshold of 0.5 are bolded.

The model was also tested on preprocessed, pre-released Live XYZ data from the San Francisco Bay area as a qualitative assessment of how performance might be hindered through large-scale food environment distribution shifts. The recall on the *Alcohol Store* class was greatly reduced by the shift from NYC to San Francisco Bay area (0.928 to 0.533), while precision was only slightly affected (0.968 to 0.871). Contrastingly, the precision on the *Grocery* class was significantly reduced (0.616 to 0.424), with the recall scores being only slightly (positively) affected (0.439 to 0.496). The *Specialty Food* class took the biggest hit, with precision and recall scores dropping from 0.867 and 0.643 to 0.394 and 0.333, respectively. Of note, the *Specialty Food* label was relatively absent from this new test set, with less than 1% (only 51 unique stores) of this dataset having a positive *Specialty Food* class (compared to more than 5.6% of the deduplicated NYC data).

### 3.2 Temporospatial patterns in the food environment

**Figure 2:**
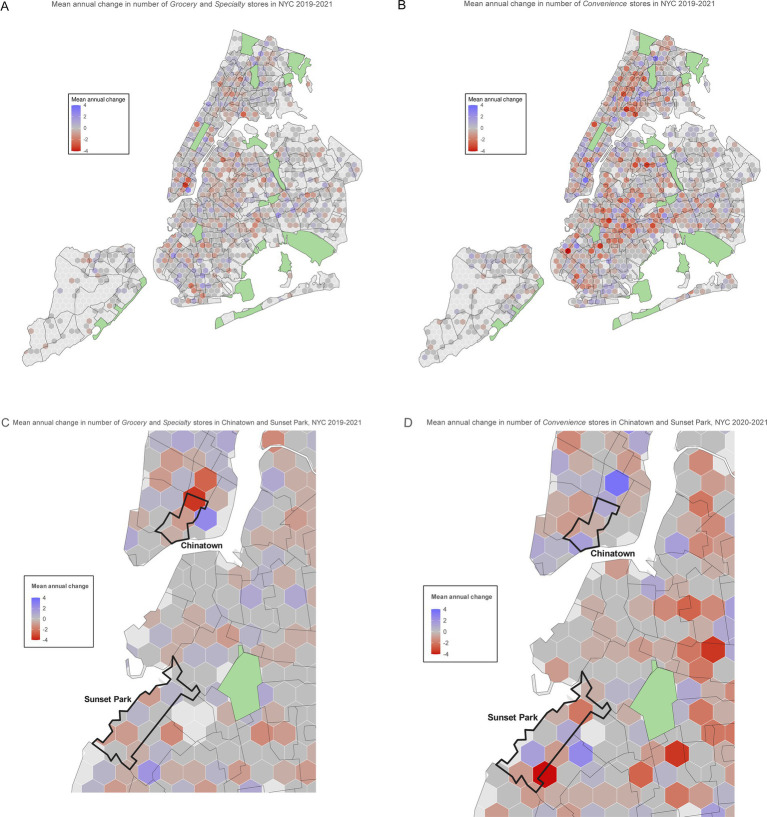
NYC maps for mean annual change in Grocery and Specialty Stores and Convenience Stores, 2019-2021. Hexbin size: 2640 ft. with overlay border 2010 NTAs **(A)** NYC Grocery and Specialty Stores: Mean businesses per hexbin, 3.7 (*SD*=0.55) **(B)** NYC Convenience Stores: Mean businesses per hexbin, 7.4 (*SD*=0.82) **(C)** Chinatown and Sunset Park Feature - Grocery and Specialty Stores: Mean businesses per hexbin, 3.7 (*SD*=0.55) **(D)** Chinatown and Sunset Park Feature - Convenience Stores: Mean businesses per hexbin, 7.4 (*SD*=0.82).

To demonstrate the utility of the classifier, the model was used to infer results from the NYSAM administrative dataset covering the period from January 2012 to December 2023. Using the model to classify stores citywide on top of geospatialized administrative data provided an opportunity to explore temporospatial trends. The churn of *Grocery, Specialty,* and *Convenience* stores were contrasted to provide a static view of a longitudinal process (openings and closings). Figure 2 displays the variability in the average churn of stores across NYC from 2019 to 2021. For each polygon, the mean annual change is represented by a divergent color scale ranging from purple (increased average number of store types) to gray (no change in numbers of store types) to red (average loss of store types). While fewer in number, the longitudinal map of the *Grocery* and *Specialty Foods* stores (Figure 2A) indicates greater segment stability than *Convenience* stores (Figure 2B). The loss of *Grocery* stores documented in the study by Yi et al. is apparent in NYC’s Chinatown in Manhattan (Figure 2C) and, to a lesser degree, in Sunset Park in Brooklyn (Figure 2D) for the period of years that span the COVID-19 pandemic and approximate Yi et al.’s study frame (Yi et al., 2022).

### 3.3 Data quality enhancements for administrative food inspection data

**Figure 3:**
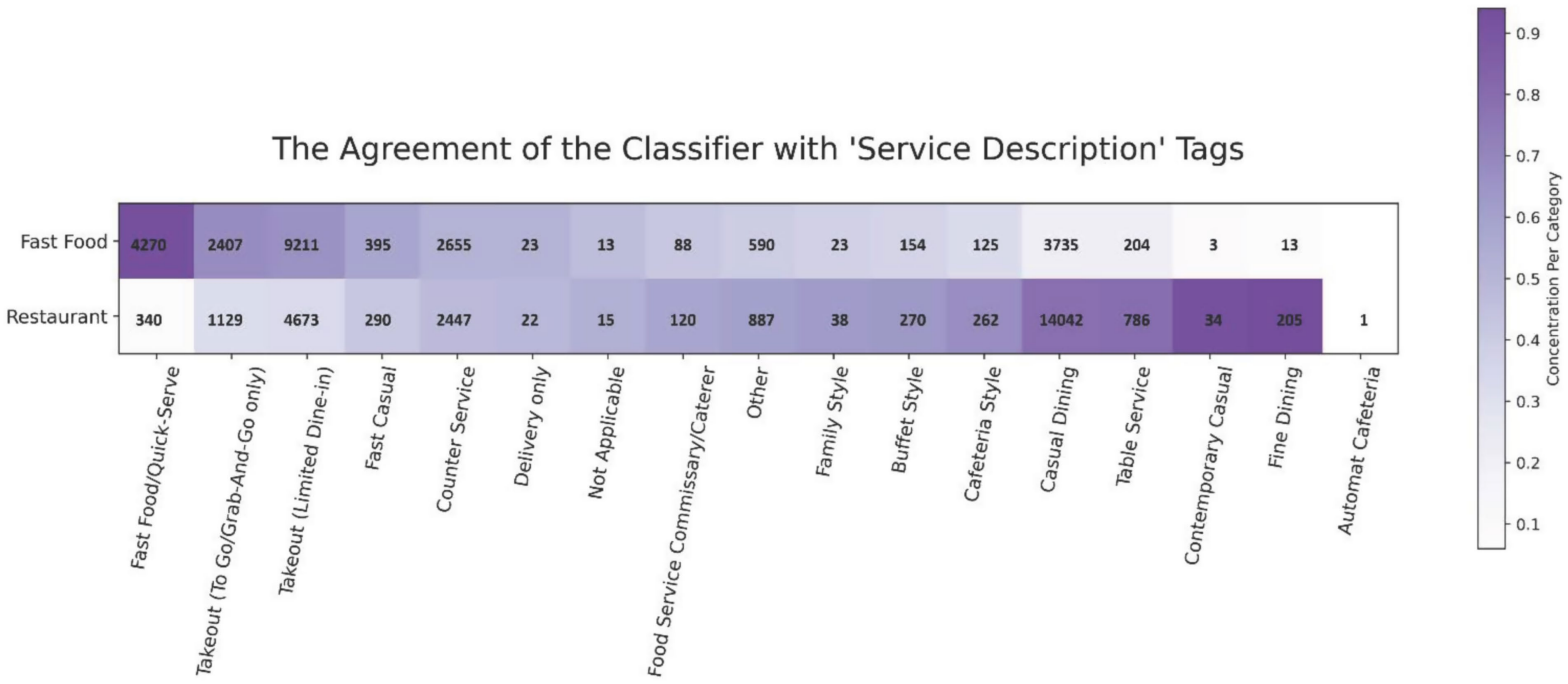
Heatmap of service description tag frequency in the restaurants dataset as compared to the fast food and restaurant classifier labels. Counts are overlayed for clarity.

The model was also applied to the DOHMH Restaurant’s dataset to demonstrate its utility for the alternate aim of DQE, restricting attention to the dataset variables service description and venue. To visualize the agreement between the classifier labels and the service description tags, a heatmap with cell counts was generated (Figure 3). The service description variable consisted entirely of the 17 tags displayed on the x-axis and the classifier’s labels of either *Fast Food* or *Restaurant* on the y-axis. Food stores labeled either *Fast Food* or *Restaurant* accounted for 73% of the data. The remaining food stores within the Restaurant’s dataset were primarily classified as *Sweets and Desserts* or *Juice/Coffee Shops* and were not included in the analysis to avoid unwanted overlap. For example, a bubble tea store might have the service description tag ‘Takeout (To Go/Grab-And-Go only),’ but by the definition of our food retail classification, it should only be labeled as *Juice/Coffee Shops.* Descriptors coinciding with a higher contrast between the *Fast Food* and *Restaurant* labels were more concentrated toward the edges of the heatmap, with those appearing to align with *Fast Food* placed on the left and *Restaurant*-type descriptors placed on the right side of the *x*-axis, except for ‘Automat Cafeteria’ which had only 1 use.

**Figure 4:**
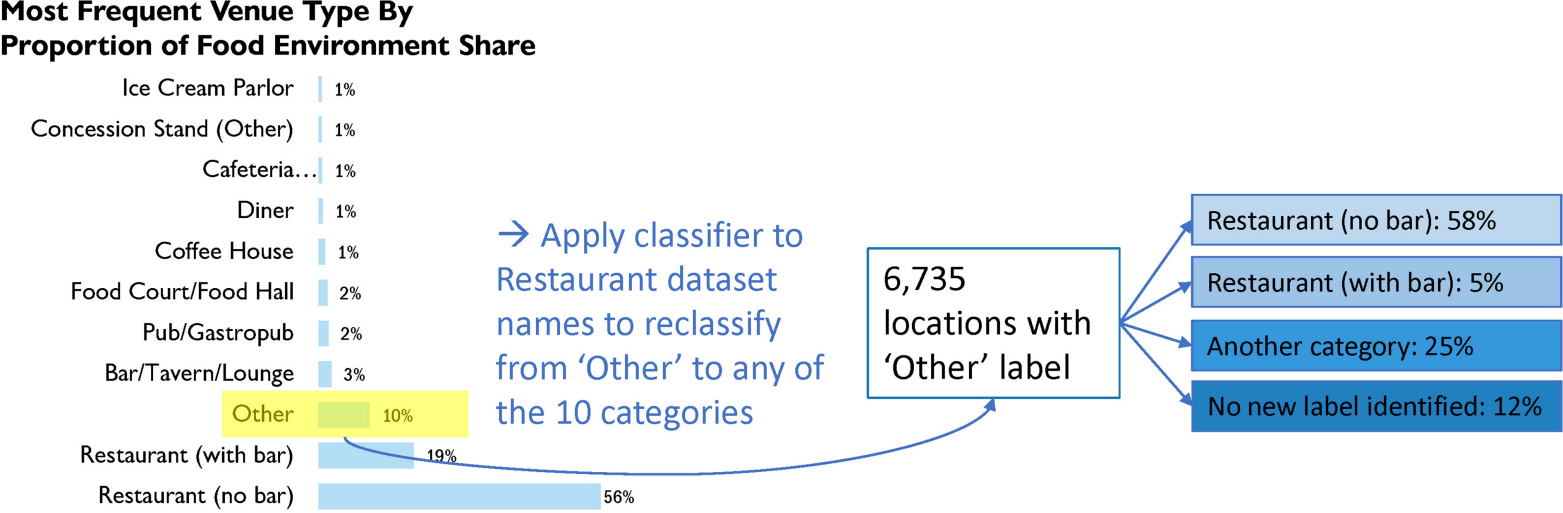
Frequencies of venue tags in the restaurant dataset: the 11 most frequent of the 41 tags belonging to this variable.

The venue variable consisted of very many (41) different venue type tags, most of which were applied with negligible frequency, the most frequent of which were listed in Figure 4. The majority of the data were assigned the tags ‘Restaurant (no bar)’ (56%), ‘Restaurant (with Bar)’ (19%), and ‘Other’ (10%). The remaining 14.4% were concentrated around tags that fit into the *Alcohol Bar* or *Juice/Coffee Shop* classes. For example, the tags ‘Bar/Tavern/Lounge’, ‘Pub/Gastropub’, and ‘Coffee House’ took up 5.9% of the venue assignments (see Figure 4). Specific to the ‘Other’ venue tags, we found that 58% of these the model inferred to belong to *Restaurants* or *Fast Food* and not to *Alcohol Bars*, indicating membership of a majority of the ‘Other’ venue tags to the overall majority assigned tag ‘Restaurant (no bar).’ A total of 5% of the ‘Other’ venue-tagged stores were inferred to belong to the *Restaurant or Fast Food* class and *Alcohol Bar* simultaneously, indicating membership to ‘Restaurant (with Bar).’ The remaining 37% of the ‘Other’ data was not labeled by our classifier as either a sit-down or fast-food restaurant. Approximately 12% of the ‘Other’ data did not receive any label at all.

## 4 Discussion

This study intended to provide a first pass at categorizing public datasets by way of a food environment classification tool. Using the store name alone, the classifier had a good performance on the test data (F1_micro_, F1_macro_ > 0.7). Per category performance was highest for *Alcohol, Convenience, Discount,* and *Fast Food* stores (F1 > 0.7). Performance in the *Grocery* store category, while still fair, was the lowest (F1 = 0.49); higher precision (0.616) and lower recall (0.439) for this category suggest *Grocery* stores were more difficult to detect using name alone, but for those that were detected, there is moderate confidence in the classification.

Known temporospatial patterns were approximated in the longitudinal inference dataset. *Grocery* and *Specialty Food* store closures from 2019 to 2021 were detected in previously identified NYC neighborhoods of Chinatown and Sunset Park (Yi et al., 2022). Notably, *Convenience* stores increased in the NTA adjacent to Chinatown (East Village, 2D) during the same time frame grocery and specialty stores were closing (2C), suggesting a shift in food acquisition dynamics. While not a direct comparison of store counts, the coarse estimates of food environment churn demonstrate the ability of the model to provide reasonable first-pass guesses even for *Grocery* and *Specialty Food* stores. High variance in *Convenience* store counts across polygons is also well reflected in the divergent color scheme shown in the longitudinal maps.

For DQE on the DOHMH Restaurants dataset, the classifier provided potential as a quality check of the service description variable and an imputation tool for the venue variable. With regard to the latter variable, the high recall (high sensitivity) scores across the *Restaurant* and *Fast Food* classes (as shown in Figure 1) reinforced confidence that the model was not errantly missing actual restaurants present in the dataset. Of the stores that were classified as *Restaurants* or *Fast Food*, the model agreed with the Restaurant’s Fast Food and Sit-down Restaurant tags more than 70% of the time. For restaurants with more vague or opaque service tags (e.g., ‘delivery only’, ‘other’, ‘caterer’, and ‘not applicable’), the classifier correctly did not assign those locations to either *Fast Food* or *Restaurant* classes, whereas for services such as ‘fine dining’ and ‘fast food,’ the model labels corresponded remarkably well. A value-add for an analyst tasked with cleaning this data would be to manually inspect discrepancies between the service description taxonomy and the labels assigned by the model. For example, inspecting the overlap between what the classifier determined to be *Fast Food*, but received the ‘fine dining’ tag.

For the venue variable, in instances where the retail type was assigned to the “Other” category (approximately 10% of all records), the classifier detected a majority (63%) of these to belong to the two most commonly occurring venue tags, “Restaurant (no bar)” and “Restaurant (with bar).” Of the remaining 37% of the data, the classifier determined a large amount (20% of the “Other” category overall) as belonging to the *Sweets and Desserts* or *Juice/Coffee* shop classes. This, in conjunction with the frequencies of tags across this variable, partially shown in Figure 3, gives empirical support for a restructuring of the taxonomy of venue types. That is, a coarser bucketing of venue types should make for more useful venue tagging of stores in the restaurant dataset. The use of this tool in datasets such as the Restaurant inspection database can further enhance the understanding of the food environment in operational planning and inspections.

Finally, with regard to our assessment of model performance on labeled food environment data from the San Francisco Bay area, the observed reduction of recall on the *Alcohol Store* class coupled with a corresponding reduction of precision in the *Grocery* class was anticipated due to the unrestricted sale of alcohol products in grocery stores. To be sure, the presence of grocery stores in the Bay area that sell a full range of alcohol products implied the multi-label *Alcohol Store* and *Grocery* would be far more frequently associated with titular grocery stores. Correspondingly, the presence of stores having a majority of their offerings as alcohol products yet still selling some groceries resulted in data containing the label *Alcohol Store* without *Grocery* (the latter being mostly replaced by *Convenience*), substantially reducing the precision on the *Grocery* class. As mentioned above, the *Specialty Food* class was unusually absent from this new test set. We attributed the significant reduction in precision in this class in part to noise in the source.

### 4.1 Previous research in food environment classification

Thorough enumeration of food retailers is a well-documented bottleneck in the process of producing a validated dataset for (secondary) food environment applications (Paquet et al., 2008; Hosler and Dharssi, 2010; Lake et al., 2010; Fleischhacker et al., 2013; Liese et al., 2013; Caspi and Friebur, 2016; Wong et al., 2017). The gold standard for enumeration is ground-truthing; however, this is cost- and time-prohibitive at scale for NYC researchers and policymakers (Powell et al., 2011). Improvements to street-level imagery of public spaces databases have begun to enrich data quality (Hirsch et al., 2021) and shrink burdens of the ground-truthing process (e.g., Google-truthing), with gains in efficiency compared to manual, in-person methods (Pliakas et al., 2017). Cohen et al. explicitly describe 70% efficiency gains by processing 15–20 addresses per hour using Google-truthing methods compared to in-person methods (Cohen et al., 2020). Efficiency gains receive a substantial boost from using the classifier described in this study, scaling with available compute resources.

Furthermore, the performance of our model in the test set and the ability to recapitulate known geospatial patterns in the NYSAM inference data reflect not only a powerful model but a very carefully curated, ground-truthed training dataset. Leveraging the strengths of privately collected and licensed data allowed for the deployment of publicly available state-of-the-art models in a government setting. While the validation of the longitudinal dataset will present challenges to scale, the classifier provided a time-saving, intuitive mechanism to classify an open-access, unlabeled food store dataset.

### 4.2 Limitations of interpretation

*Grocery* stores were the most challenging to classify for the model with moderate precision (0.616) but fair recall (0.439). Again, higher precision with lower recall suggests *Grocery* stores are not well detected by name alone, but there is moderate confidence in the *Grocery* stores that are identified. New Yorkers may require additional visual cues beyond the store name alone to make this determination (e.g., the presence of a fruit stand by the entry or store window advertising), especially for some of the smaller corner grocery stores included in the *Grocery* category.

Another limitation is that forms of noise present in administrative datasets are likely to persist through model inference and deleteriously affect the use of the data for analysis. For example, a steep volume change in the number of stores counted in the NYSAM database occurred in 2017, at the same time that the NYSAM upgraded the data storage systems. To minimize bias from the noise, we limited our analysis to years with consistent data system use that also overlapped with the comparison study period (2019–2021).

The training data represent a contemporary snapshot of the NYC food environment; hence, memorized stores (chains) that existed and closed before (or opened after) the snapshot will be challenging to longitudinally classify. In the absence of historical or future training data, addenda to the training data can address this issue for known unknowns (historical) or new chains as they are established.

While the training datasets used to build this model are curated and ground-truthed by human surveyors, the methodology by which stores were classified by the surveyors depended on subjective assessment of storefronts, not a result of how people use the store. As a result, we cannot rule out any biases in the classification.

### 4.3 Context of food environment classification

The longitudinal maps of food environment processes anticipate a temporospatial master dataset wherein future geospatial analyses of food store types can occur, addressing an outstanding need for epidemiologic study and informed policy-making at the NYC Health Department. State and local health departments bear significant responsibility for creating healthy food policies and supporting food infrastructure but are often not adequately equipped to evaluate or implement the most impactful interventions targeting diet-related chronic diseases (DRCDs) and nutrition security (NS) (Sreedhara et al., 2021). The modifying effects of food policies on DRCDs and NS via local food access have been extensively studied (Rose et al., 2010; Caspi et al., 2012), and various approaches to disrupting the theorized connections between the food environment, social norms, and unhealthy eating (Agurs-Collins et al., 2024) include mandates and restrictions on the sale of food types, economic (dis)incentives, marketing limits, information provision, and healthy default offerings (Gorski and Roberto, 2015). While interventions in the form of tax policies may disincentivize specific food choices (e.g., the sugar-sweetened beverage tax in NYC), the replacement of the policy-targeted foods with healthy, affordable, accessible, and culturally appealing alternatives requires direct changes to the local food environment and community buy-in and contributes to the persistence of the problem (e.g., turnover of a convenience store into a fruit stand) (Braid et al., 2022). Implementation science methodology and resources to evaluate the policy-driven interventions on the food environment on population DRCD and NS health outcomes is a crucial step to determining evidence-based, sustainable healthy food policies (Cohen, 2022). Moreover, as DRCDs develop over a period of exposure, the exposures must also be longitudinally defined and consistently categorized (Hirsch et al., 2021). The data required to conduct such evaluations must have categorized food store types; the methods provided in this paper facilitate this categorization process in unlabeled administrative data commonly available to health departments.

Retail segment churn is an important measure of the food environment and the interventions upon it; its measure should be considered a downstream aim facilitated by our novel data product that provides consistent, longitudinal classification and customizable geographic extents to minimize the impact of MAUPs.

### 4.4 Future directions

A closer approximation to a human-level food environment enumeration would be a natural next step toward these downstream aims. Further enhancing efficiency gains from street-level imagery, the use of multi-modal algorithms such as vision-language models (VLMs) to analyze images of food stores could also be developed for enumeration (Dai et al., 2024; Zhang et al., 2024). At the time of this paper, state-of-the-art open-weight VLMs now have OCR capabilities that would not only be able to extract store names from a storefront image but also additional data such as descriptions of services (e.g., bottle recycling and lotto) and purely visual displays (e.g., posters of products the store sold with associated prices and targeted advertisements) (Laurençon et al., 2024; Wei et al., 2024). Even more general information, such as apparent storefront size, could potentially be learned by modern VLMs. The total of this additional information would guarantee, given a sufficient amount of training data, that a VLM food store classifier would have lower generalization error than the model we have developed. A major hurdle to this approach would be procuring a dataset consisting of storefront images for inference. For example, if only point-geographic data is associated with a food store, intermediate navigation on an interactive panoramic view of streets to capture the correct image of a target storefront would be required.

Finally, further cleansing of the inference data could be undertaken should future studies require a longitudinal food environment dataset with greater accuracy for more granular analyses. Analyses of churn of food retail types, population-based health outcomes, and spatial social polarization could be easily incorporated for research or policy analyses. However, it may be that other aspects of the food environment, such as food choices and cost, will take precedence in establishing consistent linkages to downstream diet-related health outcomes over the spatial density of food stores (Block and Subramanian, 2015). How a shopper uses a store and whether a food store can accommodate demand appear to be increasingly salient inquiries into the food environment-DRCD connection [e.g., interventions in grocery retail settings that address healthy food purchasing (Wolgast et al., 2022)].

In conclusion, this application of deep learning on administrative food data resulted in enhanced efficiency of enumerating and classifying the food environment, addressing long-standing challenges to research and operations by automating expensive manual public health processes using open-access tools and data. Accomplishment of this heavily depends on the use of foundation models in a government setting. Furthermore, longitudinal and consistent assignment of meaningful categories that represent targets for intervention upon health outcomes and the use of modern geospatial analytics to facilitate rapid analytics further advance public health.

## Data Availability

The datasets presented in this article are not readily available because the data use agreement for the training data prevents the authors from disseminating the inferenced data or the final model to prevent infringement of legal agreements. The authors are not permitted to accommodate requests to access the datasets.

## Footnotes

^1^ https://www.foodenvironmentdirectory.com/

^2^Formally, we mean that such algorithms have low Kolmogorov complexity.

^3^Bert-large-uncased: https://huggingface.co/google-bert/bert-large-uncased

^4^TensorFlow early stopping: https://www.tensorflow.org/api_docs/python/tf/keras/callbacks/EarlyStopping
